# Mitochondrial Inorganic Polyphosphate (polyP) Is a Potent Regulator of Mammalian Bioenergetics in SH-SY5Y Cells: A Proteomics and Metabolomics Study

**DOI:** 10.3389/fcell.2022.833127

**Published:** 2022-02-17

**Authors:** Mariona Guitart-Mampel, Pedro Urquiza, Fausto Carnevale Neto, James R. Anderson, Vedangi Hambardikar, Ernest R. Scoma, Gennifer E. Merrihew, Lu Wang, Michael J. MacCoss, Daniel Raftery, Mandy J. Peffers, Maria E. Solesio

**Affiliations:** ^1^ Department of Biology, Rutgers University, Camden, NJ, United States; ^2^ Northwest Metabolomics Research Center, Department of Anesthesiology and Pain Medicine, University of Washington, Seattle, WA, United States; ^3^ Musculoskeletal and Ageing Science, Institute of Life Course and Medical Sciences, University of Liverpool, Liverpool, United Kingdom; ^4^ Center for Computational and Integrative Biology, Rutgers University, Camden, NJ, United States; ^5^ Department of Genome Sciences, University of Washington, Seattle, WA, United States; ^6^ Department of Environmental and Occupational Health Sciences, University of Washington, Seattle, WA, United States; ^7^ Public Health Sciences Division, Fred Hutchinson Cancer Research Center, Seattle, WA, United States

**Keywords:** mitochondria, bioenergetics, mitochondrial metabolism, OXPHOS, inorganic polyphosphate, metabolomics, proteomics, SH-SY5Y cells

## Abstract

Inorganic polyphosphate (polyP) is an ancient, ubiquitous, and well-conserved polymer which is present in all the studied organisms. It is formed by individual subunits of orthophosphate which are linked by structurally similar bonds and isoenergetic to those found in ATP. While the metabolism and the physiological roles of polyP have already been described in some organisms, including bacteria and yeast, the exact role of this polymer in mammalian physiology still remains poorly understood. In these organisms, polyP shows a co-localization with mitochondria, and its role as a key regulator of the stress responses, including the maintenance of appropriate bioenergetics, has already been demonstrated by our group and others. Here, using Wild-type (Wt) and MitoPPX (cells enzymatically depleted of mitochondrial polyP) SH-SY5Y cells, we have conducted a comprehensive study of the status of cellular physiology, using proteomics and metabolomics approaches. Our results suggest a clear dysregulation of mitochondrial physiology, especially of bioenergetics, in MitoPPX cells when compared with Wt cells. Moreover, the effects induced by the enzymatic depletion of polyP are similar to those present in the mitochondrial dysfunction that is observed in neurodegenerative disorders and in neuronal aging. Based on our findings, the metabolism of mitochondrial polyP could be a valid and innovative pharmacological target in these conditions.

## 1 Introduction

Inorganic polyphosphate (polyP) is an evolutionary conserved polymer, which is wide spread across all studied organisms, from bacteria to mammals (Kornberg et al., 1999; Kulakovskaya et al., 2010). It is composed of multiple monomers of orthophosphate linked by high-energy phosphoanhydride bonds, similar to those present in ATP (Kornberg et al., 1999). The concentration and length of polyP has shown to be variable in different subcellular locations and organisms (Kumble and Kornberg, 1995). In mammalian cells, polyP is usually formed by a couple of hundred monomers of orthophosphate, and it is found within the micromolar range (Kumble and Kornberg, 1995). The metabolism of polyP in these cells still remains poorly understood, even though a study showing the effects of the mitochondrial F_0_F_1_-ATP synthase in the synthesis and degradation of the polymer in the presence of mitochondrial respiration substrates and phosphates has been recently published (Bayev et al., 2020). However, additional pathways independent of the ATP synthase are probably also involved in the metabolism of polyP in mammalian cells (Borden et al., 2021; McIntyre and Solesio, 2021; Patro et al., 2021). In bacteria and yeast, the metabolism of the polymer is well-described. For example, it is known that polyP is converted into inorganic phosphate by the exopolyphosphatase (PPX) enzyme (Kornberg et al., 1999; Kulakovskaya et al., 2010).

PolyP has been proposed as a key molecule in the cellular stress response in a wide range of organisms, including mammals (Judge and Leeuwenburgh, 2007; Paradies et al., 2010; Armstrong et al., 2018). Accordingly, polyP has an important regulatory role in many cellular processes that are commonly dysregulated in neurodegenerative disorders, such as generation of reactive oxygen species (ROS), inflammation, apoptosis, energy metabolism, calcium signaling, and protein homeostasis (Muller et al., 2009; Kim et al., 2011; Morrissey et al., 2012; Solesio et al., 2016a; Amodeo et al., 2017; Angelova et al., 2018; Yoo et al., 2018; Maiolino et al., 2019; Solesio et al., 2020). In mammalian cells, polyP has been found in the cytoplasm, the nucleus, associated with membrane proteins (Kumble and Kornberg, 1995), in the extracellular space (Suess et al., 2017), and in different organelles such as acidocalcisomes and mitochondria, where the levels of the polymer correlate with those of ATP (Abramov et al., 2007; Solesio et al., 2016a). In fact, polyP synthesis is dependent on the metabolic status of the cell (Pavlov et al., 2005; Pavlov et al., 2010). Moreover, we have already demonstrated the role of polyP in the regulation of the intra-mitochondrial levels of free calcium, which are closely related to mitochondrial metabolism and ATP generation (Solesio et al., 2016b; Solesio et al., 2020). Indeed, intra-mitochondrial calcium signaling activates mitochondrial dehydrogenases, which leads to the increased levels of NADH and ATP (McCormack et al., 1990), present in stress response and in aging (Baltanas et al., 2013; Picard et al., 2018).

Mitochondrial function, including oxidative phosphorylation (OXPHOS) regulation, is especially relevant to high-energy cells, such as neurons (Zheng et al., 2016). Changes in energy metabolism can compromise brain function and be components in the etiopathology of multiple diseases, including neurodegenerative disorders, such as Parkinson’s (PD) and Alzheimer’s disease (AD) (Lin and Beal, 2006; Schon and Przedborski, 2011; Koopman et al., 2013). Moreover, increased ROS production, a well-known consequence of dysfunctional mitochondrial bioenergetics, is also a common aspect of neurodegeneration (Kudryavtseva et al., 2016; Singh et al., 2019). Furthermore, our research group has recently investigated the impact of mitochondrial polyP in the regulation of mammalian OXPHOS. By using HEK293 cells that are enzymatically depleted of mitochondrial polyP (MitoPPX), we demonstrated a significant shift from OXPHOS to glycolysis, globally affecting mitochondrial physiology (Solesio et al., 2021). MitoPPX cells are created by the stable transfection of Wt cells with a plasmid containing the sequence for the mitochondrial expression of the exopolyphosphatase enzyme (PPX), which is the enzyme in charge of the degradation of polyP in yeast. The homolog of the PPX enzyme in mammalian cells still remains unknown. Previous studies have shown that the effects induced by transfection with MitoPPX are due to the elimination of polyP and not to the transfection procedure or other effects of PPX on mammalian cellular physiology (Abramov et al., 2007; Seidlmayer et al., 2012; Seidlmayer et al., 2015).

Here, we conducted a comprehensive proteomics and metabolomics study of the role of mitochondrial polyP in SH-SY5Y cells, a cellular model widely used for experimental research in the field of neurodegeneration (Xicoy et al., 2017). Our goal is to better understand the contribution of mitochondrial polyP to the molecular mechanisms that regulate cellular bioenergetics. To achieve this, we used Wild-type (Wt) and MitoPPX SH-SY5Y cells. Proteomics and metabolomics approaches were conducted to identify phenotypic differences between the cell lines, particularly focused on mitochondrial metabolism and bioenergetics. Our data suggested that the enzymatic depletion of mitochondrial polyP has a deleterious impact on various metabolic pathways, including mitochondrial bioenergetics, where several subunits of the Electron Transfer Chain (ETC) as well as metabolites, are altered. These integrated proteomics and metabolomics profiles could bring new insights into the specific role(s) of mitochondrial polyP in mammalian cells, as well as contribute to finding new therapeutic approaches using mitochondrial polyP as a valid target against dysfunctional organelle in human pathologies.

## 2 Materials and Methods

### 2.1 Reagents

Dulbecco’s Modified Eagle medium (DMEM):F12; 4′,6-diamino-2-phenylindole (DAPI); penicillin-streptomycin; G418; lipofectamine; trypsin; alkaline phosphatase; Pierce BCA protein assay kit; Pierce ECL western blotting substrate; phosphoric acid; heat-inactivated fetal bovine serum (FBS); acetonitrile (ACN); methanol; ammonium acetate; Pierce Halt protease and phosphatase Inhibitor Cocktails; and acetic acid Optima LC-MS grade were purchased from Thermo Fisher Scientific (Waltham, MA, US). Phosphate-Buffered Saline (PBS); *β*-mercaptoethanol; tris(hydroxymethyl)-1,3-propanediol hydrochloride (TRIS-HCl); glycerol; Bovine Serum Albumin (BSA); phenylmethylsulfonyl fluoride (PMSF); Tween-20; triethylammonium bicarbonate buffer (TEAB); dimethyl sulfoxide (DMSO); iodoacetamide (IAA); yeast enolase protein; and potassium chloride were obtained from Sigma-Aldrich (San Louis, Missouri, US). All the materials and reagents used in the Western Blotting experiments, including secondary antibodies, polyvinylidene (PVDF) membranes, fat-free milk, protein ladders, and polyacrylamide-precast gels were obtained from BioRad (Hercules, California, US). Primary antibodies anti-OXPHOS, anti-PPX and anti-β-actin were obtained from Abcam (Cambridge, United Kingdom), and anti-eGFP from Cell Signaling (Danvers, MA, US). Deionized water was obtained using an 18 MΩ Milli-Q from EMD Millipore Corporation (Billerica, Massachusetts, US).

### 2.2 Cell Growing and Maintenance

Wt SH-SY5Y cells were obtained from the American Type Culture Collection (Manassas, Virginia, US), and grown following the instructions of the provider, as previously done (Solesio et al., 2012; Solesio et al., 2013; Fossati et al., 2016; Angiulli et al., 2018; Solesio et al., 2018). Specifically, we used DMEM:F12 media, supplemented with 20 units/mL penicillin-streptomycin and 10% (v/v) heat-inactivated fetal bovine serum (FBS). Cells were grown in a humidified cell culture incubator, under a 5% CO_2_ atmosphere at 37°C until around 80% optimal confluence was reached, as previously reported (Solesio et al., 2012; Solesio et al., 2013). MitoPPX cells were generated adapting the protocol from (Solesio et al., 2020) to SH-SY5Y neuroblastoma cells. 50,0000 cells were plated in 6-well plate. 24 h later, the cells were transiently transfected using lipofectamine and a DNA construct containing the sequence for the mammalian expression of a mitochondrial targeting sequence (MTS), green fluorescent (eGFP) protein, and PPX (Solesio et al., 2020). After 24 h, 0.5 mg/mL of geneticin (the selection antibody) were added to the cells for 2 weeks. Subsequently, individual clones were selected, transferred into a 96-well plate and amplified. MitoPPX cells were grown and maintained in the presence of geneticin to assure the expression of the construct.

### 2.3 PPX Enzymatic Assay

The binding between polyP-DAPI shifts the wavelength of emission of DAPI up to 550 nm (Aschar-Sobbi et al., 2008). Therefore, this is a widely used method to assay the presence of polyP in various organisms, including mammalian cells (Abramov et al., 2007; Aschar-Sobbi et al., 2008). Using this principle, the enzymatic activity of MitoPPX cells was assayed following the protocol previously published in (Solesio et al., 2021).

### 2.4 Protein Extraction and Quantification

Cells were plated and grown to 90% confluency. Afterwards, cells were scrapped on ice-cold PBS 1x, and centrifuged at 1,000 rpm for 5 min at 4°C. Pellets were re-suspended in lysis buffer (300 mM NaCl, 50 mM Tris-HCl pH 7.5, 1% TritonX-100) and shaken for 15 min at 4°C. Cell suspensions were centrifuged at 13,000 rpm for 5 min at 4°C, and supernatants were directly frozen at–80°C. Protein content was measured through the bicinchoninic acid colorimetric (BCA) assay following manufacturer’s instructions. Aliquots of the samples were prepared and stored at -80°C for further Western Blotting validation.

### 2.5 Western Blotting

Western Blotting analyses were conducted as previously published (Baltanas et al., 2013; Liu et al., 2019; Castro et al., 2020; Khong et al., 2020). 20 µg of protein per sample was separated using 12% Mini-Protean TGX Precast Gel, and transferred into PVDF membranes. Non-specific protein binding was blocked using 5% fat-free milk and 0.1% Tween-20 in PBS 1x for 1 h. Membranes were then hybridized with the specific primary antibodies overnight at 4°C (PPX 45 kDa; eGFP 25 kDa; CV-ATP5A 54 kDa; CIII-UQCRC2 48 kDa; CII-SDHB 29 kDa; CIV-COXII 22 kDa; CI-NDUFB8 18 kDa; all of them at 1:1,000 dilution). The levels of protein expression were normalized using *β*-actin content (47 kDa; 1:1000). The signal was detected using Piece ECL Substrate kit following manufacturer’s instructions and the Gel Doc XR Image System from BioRad (Hercules, California, US). Finally, the intensity of the signal was quantified by densitometry analysis using ImageJ software from NIH (Bethesda, Maryland, US).

### 2.6 Proteomics Assay

Wt and MitoPPX cells were scraped, washed with cold PBS, and immediately preserved at–80°C (five replicates, collected on two independent days). Cells were shipped overnight on dry ice for further protein profiling at the Department of Genome Science of University of Washington (Seattle, Washington, United States).

#### 2.6.1 Protein Lysis and Digestion

Cell pellets were resuspended in 100 μL of 5% SDS, 50 mM Triethylammonium bicarbonate (TEAB), 2 mM MgCl_2_, and 1x Pierce Halt protease and phosphatase inhibitor cocktail, vortexed and briefly probe sonicated. Protein concentration was measured with a BCA assay. Homogenate of 50 ug was added to a process control of 800 ng of yeast enolase protein, which was then reduced with 20 mM DTT, and alklyated with 40 mM of IAA. Lysates were then prepared for S-trap column (Protifi, Long Island, New York, US), binding by the addition of 1.2% phosphoric acid and 350 μL of binding buffer (90% methanol, 100 mM TEAB). The acidified lysate was bound to the column incrementally, followed by three wash steps with binding buffer to remove SDS, three wash steps with 50:50 methanol:chloroform to remove lipids, and a final wash step with binding buffer. Trypsin (1:10) in 50 mM TEAB was then added to the S-trap column for digestion at 47°C for 1 h. Hydrophilic peptides were then eluted with 50 mM TEAB and hydrophobic peptides were eluted with a solution of 50% acetonitrile in 0.2% formic acid. The elute was pooled, speed vacuumed and resuspended in 0.1% formic acid. A heavy labeled Peptide Retention Time Calibrant (PRTC) mixture (Pierce, Waltham, Massachusetts, US) was added to each sample.

#### 2.6.2 Proteomic Profiling by LC-MS

One μg of each sample with 150 femtomole of PRTC were loaded onto a 30 cm fused silica picofrit (New Objective, Littleton, Massachusetts, US) 75 μm column and 3.5 cm 150 μm fused silica Kasil1 (PQ Corporation, Malvern, Pennsylvania, US) frit trap loaded with 3 μm Reprosil-Pur C18 (Dr. Maisch, Ammerbuch, Entringen, Germany) reverse-phase resin analyzed with a Thermo Easy nano-LC 1200. The PRTC mixture was used to assess quality of the columns before and during analysis. Four of these quality control runs were analyzed prior to any sample analysis and then after every six to eight sample runs, another quality control run was analyzed.

Buffer A was 0.1% formic acid in water and Buffer B was 0.1% formic acid in 80% acetonitrile. The 40-min QC gradient consists of a 0–16% B in 5 min, 16–35% B in 20 min, 35–75% B in 1 min, 75–100% B in 5 min, followed by a wash of 9 min and a 30 min column equilibration. The 110 min sample LC gradient consists of a 2–7% for 1 min, 7–14% B in 35 min, 14–40% B in 55 min, 40–60% B in 5 min, 60–98% B in 5 min, followed by a 9-min wash and a 30-min column equilibration. Peptides were eluted from the column with a 50°C heated source (CorSolutions, Ithaca, New York, US) and electrosprayed into a Thermo Orbitrap Fusion Lumos Mass Spectrometer with the application of a distal 3 kV spray voltage. For the quality control analysis, a cycle of one 120,000 resolution full-scan mass spectrum (350–2000 *m/z*) followed by data-independent MS/MS spectra on the loop count of 76 data-independent MS/MS spectra using an inclusion list at 15,000 resolution, AGC target of 4e5, 20 s maximum injection time, 33% normalized collision energy with a 8 *m/z* isolation window. For the sample digestion, first a chromatogram library of six independent injections is analyzed from a pool of all samples within a batch. For each injection, a cycle of one 120,000 resolution full-scan mass spectrum with a mass range of 100 *m/z* (400–500 *m/z*, 500–600 *m/z* … 900–1,000 *m/z*) followed by data-independent MS/MS spectra on the loop count of 26 at 30,000 resolution, AGC target of 4e5, 60 s maximum injection time, 33% normalized collision energy with a 4 *m/z* overlapping isolation window. The chromatogram library data generated from a pooled samples was used to detect proteins from individual quantitative sample runs. These individual runs consist of a cycle of one 120,000 resolution full-scan mass spectrum with a mass range of 350–2000 *m/z*, AGC target of 4e5, 100 m maximum injection time followed by data-independent MS/MS spectra on the loop count of 76 at 15,000 resolution, AGC target of 4e5, 20 s maximum injection time, 33% normalized collision energy with an overlapping 8 *m/z* isolation window. Application of the mass spectrometer and LC solvent gradients are controlled by the ThermoFisher XCalibur data system (Waltham, Massachusetts, US).

#### 2.6.3 Proteomics Data Analysis

Thermo RAW files were converted into mzML format using Proteowizard (version 3.0.20064; Palo Alto, CA, US) using vendor peak picking and demultiplexing (Amodei et al., 2019). Chromatogram libraries were created by analyzing the six narrow window gas phase fractionated runs on the pool sample using default settings (10 ppm tolerances, trypsin digestion, HCD b- and y-ions) of EncyclopeDIA (version 0.9.5) using a Prosit predicted spectra library (Gessulat et al., 2019) based on Uniprot human canonical FASTA background (april 2019) as described previously (Pino et al., 2020). Prosit library was created using the settings one missed cleavage, 33% Normalized Collison Energy (NCE), charge states of 2 and 3, m/z range of 396.4–1,002.7, and a default charge state of 3. The resulting chromatogram library empirically corrected the on-column chromatographic retention times and the product ion intensities and only included peptides detected in the pooled sample (Pino et al., 2020).

The individual quantitative analyses were analyzed using EncyclopeDIA with the Chromatogram library generated from the narrow window gas phase fractionated data. EncyclopeDIA was set to require a minimum of three quantitative ions and filtering peptides at a 1% False Discovery Rate (FDR) using Percolator 3.01 (Kall et al., 2008). The output of the EncyclopeDIA Quant Report was imported into Skyline (version 20.1.9.234; Fairfield, Ohio, US) with the human uniprot FASTA as the background proteome to map peptides to proteins. We used a library of predicted spectra and retention times. In Skyline, data was normalized to the total ion current (TIC) and unique peptides were summed to protein TAF (total area fragment) quantities. If a peptide mapped to more than one protein, Skyline selected the first protein on the list. A csv file of unique protein TAFs for each replicate was exported. The Skyline documents and raw files for DIA library generation and DIA sample analyses are available at Panorama Public (ProteomeXchange ID: PXD028185; access URL: https://panoramaweb.org/MitoPPX.url)

### 2.7 Metabolomics Assay

#### 2.7.1 Sample Preparation

Cells were prepared as for the proteomics assay (five replicates, collected on two independent days), and shipped over night on dry ice for metabolite profiling at the University of Washington’s Northwest Metabolomics Research Center (Seattle, Washington, US).

Aqueous metabolites were extracted using a protein precipitation method (Mathon et al., 2019). Cell samples were first homogenized in 200 µL purified deionized water at 4°C, and then 800 µL of methanol containing ^13^C_6_-glucose and ^13^C_2_-glutamate (reference internal standards used to monitor sample preparation) were added. Afterwards samples were vortexed, stored for 30 min at–20°C, sonicated in an ice bath for 10 min, centrifuged for 15 min at 14,000 rpm and 4°C, and then 600 µL of supernatant was collected from each sample. Lastly, recovered supernatants were dried on an Eppendorf Vacufuge (Brinkmann Instruments, Westbury, NY, US) and reconstituted in 1 ml of LC-matching solvent containing ^13^C_2_-tyrosine and ^13^C_3_-lactate (reference internal standards used to monitor instrument performance). Protein pellets that were left over from the sample preparation were used for BCA protein assay.

#### 2.7.2 Metabolite Profiling by LC-MS

Targeted LC-MS metabolite analysis was performed on a duplex-LC-MS system composed of two Shimadzu Nexera XR LC-20AD pumps, CTC Analytics PAL HTC-xt temperature-controlled auto-sampler (Shimadzu, Kyoto, Japan) and AB Sciex 6,500 + Triple Quadrupole MS equipped with ESI ionization source (AB Sciex, Framingham, MA, US) (Meador et al., 2020). UPLC pumps were connected to the auto-sampler in parallel to allow two independent chromatography separations: while one column performed separation and MS data acquisition in ESI + ionization mode, the other column was equilibrated for sample injection, chromatographic separation and MS data acquisition in ESI- mode. Samples were injected on two identical Waters XBridge BEH Amide XP analytical columns (-2.5 µm, 130 Å, 2.1 × 150 mm) (Waters Corporation, Milford, MA, US). Each chromatography separation was 18 min (total analysis time per sample was 36 min). MS data acquisition was performed in multiple-reaction-monitoring (MRM) mode. LC-MS system was controlled using AB Sciex Analyst 1.6.3 software (AB Sciex, Framingham, MA, US).

#### 2.7.3 Metabolomics Data Analysis

Measured MS peaks were integrated using AB Sciex MultiQuant 3.0.3 software. The LC-MS assay targeted 363 metabolites (plus four spiked reference internal standards). In addition to the study samples, two sets of quality control (QC) samples were used to monitor the assay performance as well as data reproducibility. One QC [QC(I)] was a pooled human serum sample used to monitor system performance over extended time and the other QC [QC(S)] was a pooled study sample, which was used to monitor data reproducibility. Each QC sample was injected per every 10-study samples. The data were well reproducible with a median CV of 5.86%.

### 2.8 Statistical Analysis

For the proteomics data, we used TIC-normalized protein TAF quantities for the downstream analysis. Data were run in two batches. There were 5,847 proteins detected in run batch1 and 5,600 proteins detected in run batch2. We included 4,232 proteins with <20% missingness in both batches for the imputation. We used a quantile regression approach for the imputation of left-censored missing data (QRILC), which has been suggested as the favored imputation method for left-censored Missing Not At Random (MNAR) data (Wei et al., 2018). This was implemented in the R imputeLCMD package. We fitted a linear model to the protein level data to detect the genotype group differences while adjusting for run batch and sample collection day in the model using the Bioconductor limma package (Ritchie et al., 2015). The limma package uses empirical Bayes moderated statistics, which improves power by ‘borrowing strength’ between proteins in order to moderate the residual variance (Smyth, 2004).

For the targeted metabolomics assay, we performed a median normalization where we adjusted the data, so all samples have the same median value of the metabolite abundance post log_2_ transformation. Only metabolites with <20% missingness and a coefficient of variation (CV) < 20% in the pooled sample QC data were included in further analyses. Out of the possible 363 metabolites that the assay could detect, 151 metabolites passed these filtering criteria, which were included in the imputation step. We used the same imputation method as described above for the proteomics data. We fit a linear model to the imputed data to detect the genotype group differences while adjusting for collection day and protein amount in the model using the Bioconductor limma package.

For the unsupervised learning, the batch effects (run batch and sample collection day) and covariates (protein amount) were removed using the limma removeBatchEffect function prior to the PCA (principal component analysis) and clustering analysis. We used MetaboAnalyst (v 5.0) to perform univariate methods (*t*-test), multivariate analysis (PCA) and hierarchical clustering (Euclidean distance and Ward’s linkage) (Pang et al., 2021). Statistical analysis and graphical representation of the data were conducted using GraphPad (San Diego, California, US), and Origins Lab (Northampton, Massachusetts, US) software.

### 2.9 Pathway Analysis

Ingenuity Pathway Analysis (IPA, Ingenuity Systems, Redwood City, California, US), was used to analyze the proteomics and metabolomics data for canonical pathways, upstream regulators, and disease and function analysis using the list of differentially expressed proteins (log_2_FC cutoff of 1.5 and FDR<0.001) and metabolites (FDR<0.05). Protein and metabolite symbols were used as identifiers. All molecules were overlaid onto a global molecular network contained in the Ingenuity Knowledge base. Networks of network-eligible molecules were algorithmically generated based on their connectivity. The functional analyses identified the canonical pathways, upstream regulators and biological functions and diseases that were most significant to the data set. A right-tailed Fisher’s exact test was used to calculate the raw *p*-values. The z-score was used to predict the activation or inhibition state of the molecules in our datasets. Canonical pathways, upstream regulators and biological functions and diseases which were likely activated (based on the pattern of differentially abundant proteins or metabolites) were presented in orange (positive z-score), those that were likely inhibited were presented in blue (negative z-score), and those with a z-score which is zero (or close to zero) or ineligible for prediction were presented in white or grey, respectively (NaN z-score).

## 3 Results

### 3.1 SH-SY5Y Cells Overexpressing the PPX Enzyme in Mitochondria Showed Decreased Levels of polyP and Affected ETC

**FIGURE 1:**
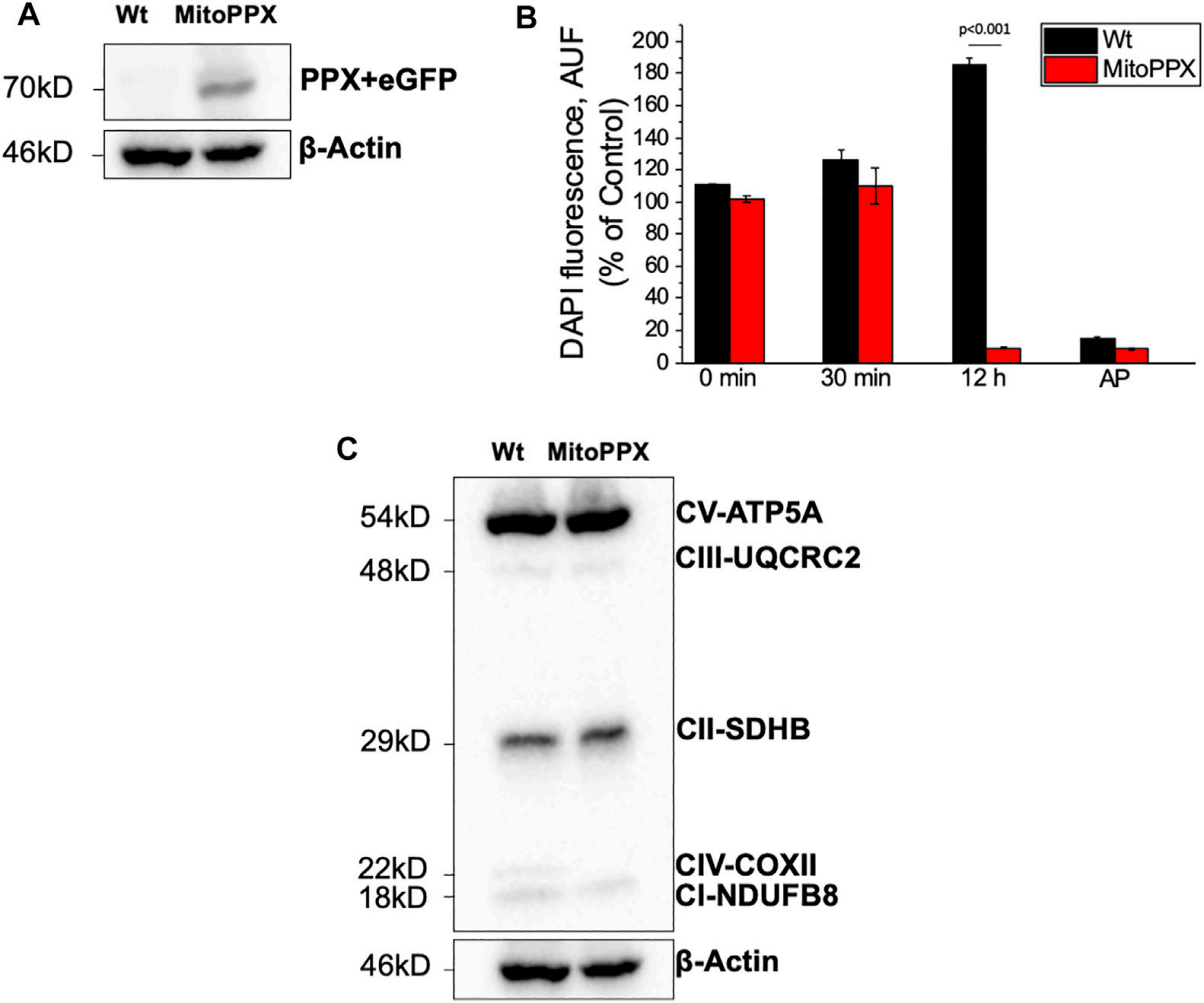
SH-SY5Y neuroblastoma cells overexpressing exopolyphosphatase (PPX) enzyme. **(A)** Western Blotting confirming the expression of PPX (70 kDa together with eGFP protein) in the MitoPPX cells. PPX antibody was used to conduct this experiment. However, the molecular weight shown is the combined molecular weight of PPX and GFP, as the GFP protein is expressed jointly with PPX. **(B)** Enzymatic assay demonstrating the activity of the PPX enzyme in our MitoPPX SH-SY5Y samples. After 12 h of treatment with exogenous polyP, the levels of the polymer were decreased in MitoPPX cells, compared to Wt (*p* < .001). These decreased levels were comparable to the levels seen in the positive control, which was conducted with alkaline phosphatase (AP), an enzyme known to degrade polyP (Lorenz and Schroder, 2001). Results are expressed as mean ± SEM of three experiments. *t*-test was used to seek for statistical analysis between groups. **(C)** Western Blotting that shows the expression of different subunits of the ETC complexes in SH-SY5Y Wt and MitoPPX samples that were shipped for metabolomics and proteomics assays (*n* = 1).

SH-SY5Y MitoPPX cells successfully expressed eGFP and the PPX enzyme, while these proteins were not observed in the Wt samples (Figure 1A). In addition, the activity of the PPX enzyme was assayed through the measurement of the cellular levels of exogenous polyP, using DAPI fluorescence as previously described (Aschar-Sobbi et al., 2008; Solesio and Pavlov, 2016). A significant decrease of cellular polyP was observed in MitoPPX cells after 12 h of incubation with exogenous polyP, when compared with Wt (Figure 1B). This indicated the functional activity of the overexpressed PPX enzyme in MitoPPX cells.

The expression of some subunits of the ETC complexes was assayed using Western Blotting in our Wt and MitoPPX SH-SY5Y cells. Decreased protein expression of NDUFB8-complex I and COXII- complex IV subunits were observed in MitoPPX cells, compared to Wt. These results will be confirmed by our data obtained from the proteomics and metabolomics assays, and they show the important role of polyP in the regulation of the function of the ETC (Figure 1C. Different exposure times of the membrane are included in Supplementary Figure S1).

### 3.2 Pathway Analysis of Proteomics and Metabolomics Data Show Significant Changes in MitoPPX SH-SY5Y Cells, Compared With Wt Cells, Including Differences in Bioenergetics

The global effects of the enzymatic depletion of mitochondrial polyP were investigated through the IPA software. We focused our studies especially on the pathways that relate to mitochondrial metabolism. Each dataset was analyzed separately and later integrated via meta-analysis. A complete summary of each IPA analysis (Proteomics, Metabolomics and Meta-analysis) can be accessed in the Supplementary Material S1.

#### 3.2 1 Differential Protein Expression

**FIGURE 2:**
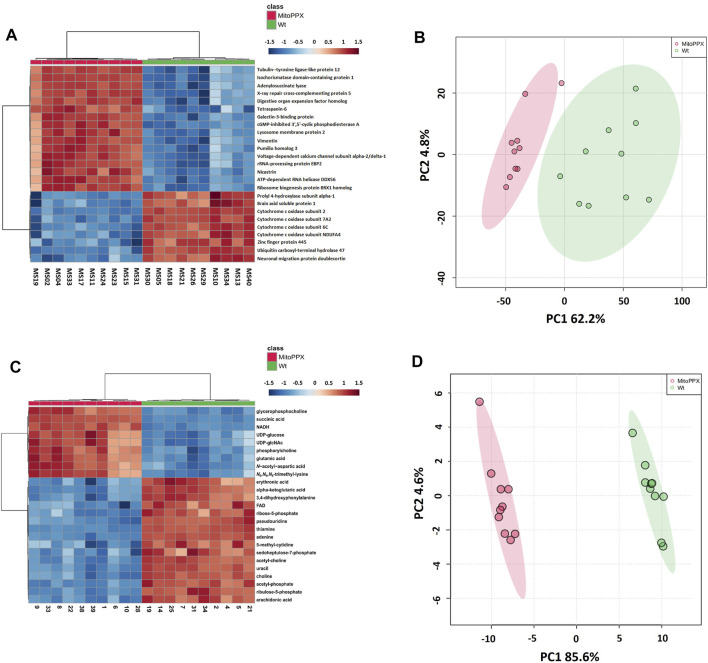
Heat maps and principal component analysis (PCA) showed that SH-SY5Y Wt and MitoPPX have different proteomics and metabolomics profiles. Heat maps identified the top 25 most significantly expressed proteins (*t*-test *p*-value<.05) **(A)** and metabolites (*t*-test *p*-value<.05) **(C)** detected in MitoPPX cells with respect to Wt cells, some of them related to the status of mitochondrial physiology. Red colors depict proteins or metabolites that were upregulated in the MitoPPX cells and blue colors depict those downregulated in the same samples. PCA identified clear separation between groups for both proteomics **(B)** and metabolomics **(D)** datasets. Shaded areas depict regions with at least 95% confidence.

Proteomics analysis led to the annotation of 4,232 proteins with less than 20% missingness in all samples. Out of 4,214 mapped proteins in IPA, we identified 405 differentially expressed proteins in MitoPPX cells compared to Wt (log2FC < 1.5 and FDR<0.001), of which 386 were upregulated, whereas 19 were downregulated. The top 25 most significantly expressed proteins with a *t*-test *p*-value<0.05 were depicted in a heat map (Figure 2A). PCA demonstrated a clear discrimination between the two groups (in red MitoPPX cells and in green Wt cells; Figure 2B).

#### 3.2.2 Differential Metabolite Abundance

Out of 363 metabolites, the metabolomics profiling in IPA resulted in the detection of 151 metabolites with less than 20% missingness in all samples, and 80 abundant metabolites were significantly different in MitoPPX compared to Wt (FDR<0.05), of which 52 were upregulated and 36 were downregulated. The top 25 most significant metabolites (*t*-test *p*-value<0.05) were shown in a heat map (Figure 2C). PCA showed two clear populations (in red MitoPPX cells and in green Wt cells; Figure 2D).

#### 3.2.3 Identification of Canonical Pathways

**FIGURE 3:**
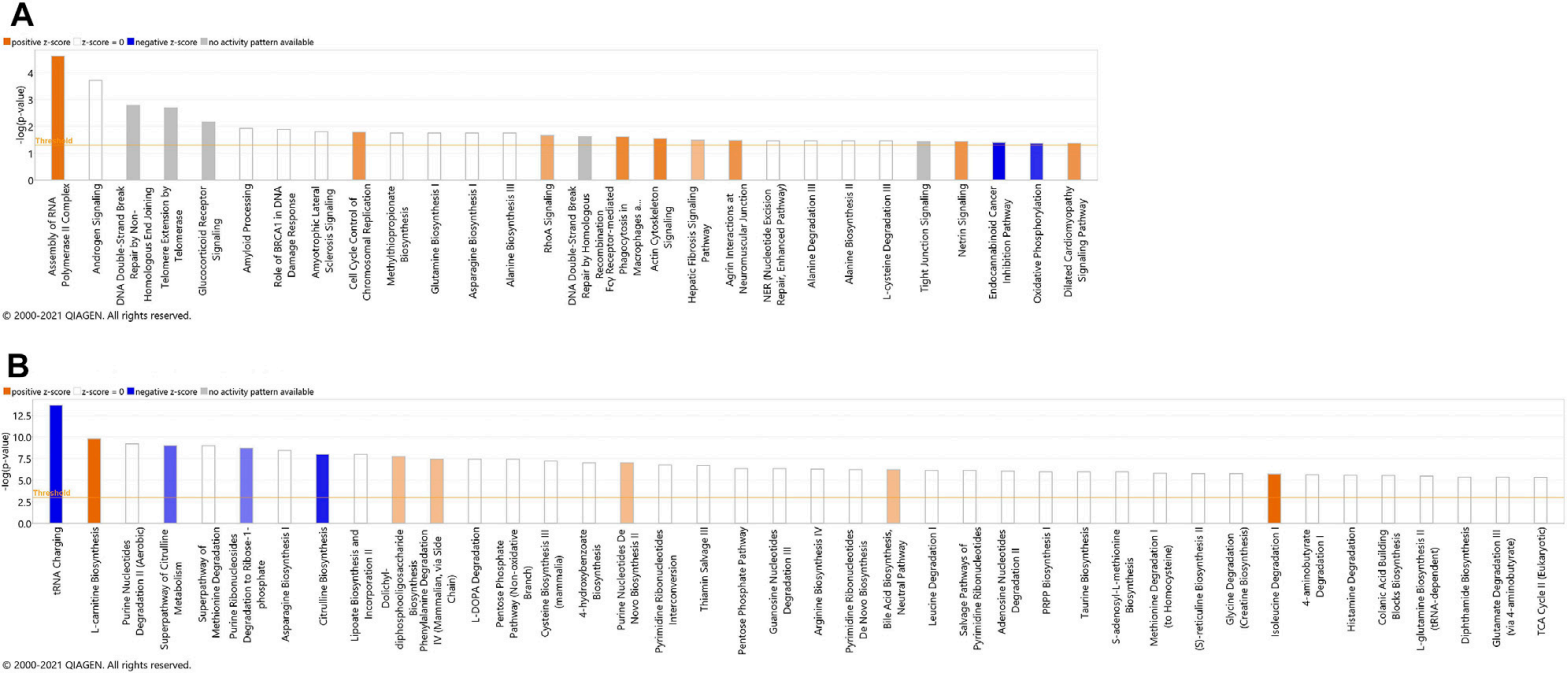
Canonical pathways identified using IPA analysis, based on proteins **(A)** and metabolites **(B)** that were identified using label-free quantification proteomics (log_2_FC≥1.5 and FDR<0.05) and metabolomics (FDR<0.05). The bars represent the significance of the canonical pathway as calculated by a right-sided Fisher’s exact test (threshold is showing raw *p*-value<.05 for proteomics and raw *p*-value<.001 for metabolomics). Therefore, the tallest bars represent the canonical pathways that are the least likely to have been identified due to molecules being in the canonical pathway by random chance. Canonical pathways which are likely activated (based on the pattern of differentially abundant proteins or metabolites) are presented in orange (positive z-score) and pathways that are likely inhibited are presented in blue (negative z-score).

The most significant dysregulated pathways in the proteomics analysis included assembly of RNA polymerase II complex (raw *p*-value<.001), androgen signaling (raw *p*-value<.001), DNA double-strand break repair by non-homologous end joining (raw *p*-value<.01), telomere extension by telomerase (raw *p*-value < .01), glucocorticoid receptor signaling (raw *p*-value<.01), and amyloid processing (raw *p*-value<.05) (Figure 3A). In the metabolomics analysis, the most significant dysregulated pathways comprised tRNA changing, l-carnitine biosynthesis, purine nucleotides degradation II–aerobic, super pathway of citrulline metabolism, and super pathway of methionine degradation (all of them raw *p*-value<0.001) (Figure 3B).

**FIGURE 4:**
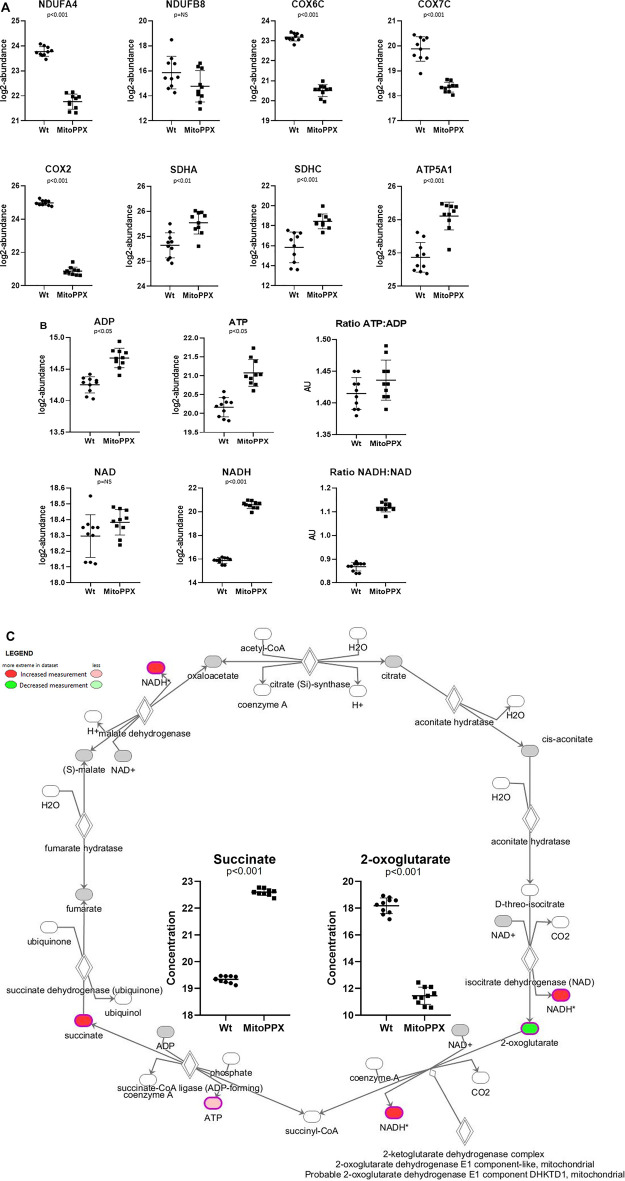
SH-SY5Y MitoPPX cells showed alterations in OXPHOS and in TCA. **(A)** Proteomics data showed decreased and increased protein expression of distinct subunits of the ETC complexes in MitoPPX cells compared to Wt. **(B)** Metabolomics data showed increased presence of different metabolites involved in mitochondrial metabolism in MitoPPX cells compared to Wt. **(C)** Metabolomics data showed alterations in the levels of succinate and 2-oxoglutarate metabolites in MitoPPX cells as well as in the levels of ATP and NADH metabolites lead to a dysfunction of TCA. Ten biological samples (five replicates, collected on two independent days) were used for each experiment. All results were expressed as mean ± SD and a right-tailed Fisher’s exact test was used to calculate the raw *p*-values.

Both proteomics and metabolomics analyses showed the differential expression of the OXPHOS pathway in MitoPPX cells, compared to Wt samples (raw *p*-value<.05 and <.001, respectively). Proteomics data indicated that the variation in OXPHOS occurred at different subunits of the ETC complexes (Table 1 and Figure 4A) which was accompanied by alterations in the abundance of ATP, ADP, NADH and NAD + metabolites, all of them directly involved in OXPHOS (Table 2; Figure 4B). These results were supported by IPA prediction of decreased OXPHOS activity in the MitoPPX cells, when compared to Wt, proposed in both proteomics (negative z-score = -1.34; Figure 3A) and metabolomics datasets (negative z-score = -1). The dysregulation of the tricarboxylic acid (TCA) cycle observed in the metabolomics data (Figure 3B), with increased succinate and decreased oxoglutarate, also suggested a global effect of polyP depletion on mitochondrial function and energetic metabolism (Figure 4C).

**TABLE 1:** List of the five altered proteins of the OXPHOS pathway in MitoPPX cells compared to Wt, identified by IPA software. ETC components showed significant differences in their expression levels (log_2_FC < 0 means decreased expression and log_2_FC > 0 means increased expression) in MitoPPX cells, compared to Wt. Alterations in these proteins allowed the IPA software to predict a decrease in the OXPHOS system. The ‘Expected’ column indicates the state that protein is predicted to have if the pathway were activated. FDR: False Discovery Rate.

Symbol	Gene Name	UniProt	Log_2_FC	FDR	Expected
COX6C	Cytochrome c oxidase subunit 6C	P09669	−2.67	2.40E-21	Up
COX7A2	Cytochrome c oxidase subunit 7A2	P14406	−2.31	1.89E-21	Up
COX7C	Cytochrome c oxidase subunit 7C	P15954	−1.54	2.78E-07	Up
NDUFA4	NDUFA4 mitochondrial complex associated	O00483	−2.00	3.82E-17	Up
SDHC	Succinate dehydrogenase complex subunit C	Q99643	2.61	3.38E-06	Up

**TABLE 2:** List of the four altered metabolites involved in the OXPHOS pathway in MitoPPX cells compared to Wt, identified by IPA software. Metabolites which were significantly different (log_2_FC < 0 means decreased expression and log_2_FC > 0 means increased expression) in MitoPPX cells compared to Wt. Alterations in these metabolites allowed the IPA software to predict a decrease in the OXPHOS system. The ‘Expected’ column indicates the state that protein is predicted to be in if the pathway were activated. FDR: False Discovery Rate.

Symbol	Human Metabolome Database (HMDB)	Log_2_FC	FDR	Expected
Adenine-riboflavin dinucleotide	HMDB01248	−1.13	7.78E-05	Up
ATP	HMDB00538	0.91	2.69E-02	Up
NADH	HMDB01487	4.70	8.55E-06	Down
Succinic acid	HMDB00254	3.26	5.61E-13	Down

#### 3.2.4 Analysis of Upstream Regulators

We performed upstream regulator analysis (URA) in IPA software to explore the potential upstream regulators of the protein and metabolite regulatory networks affected by changes in gene expression.

**FIGURE 5:**
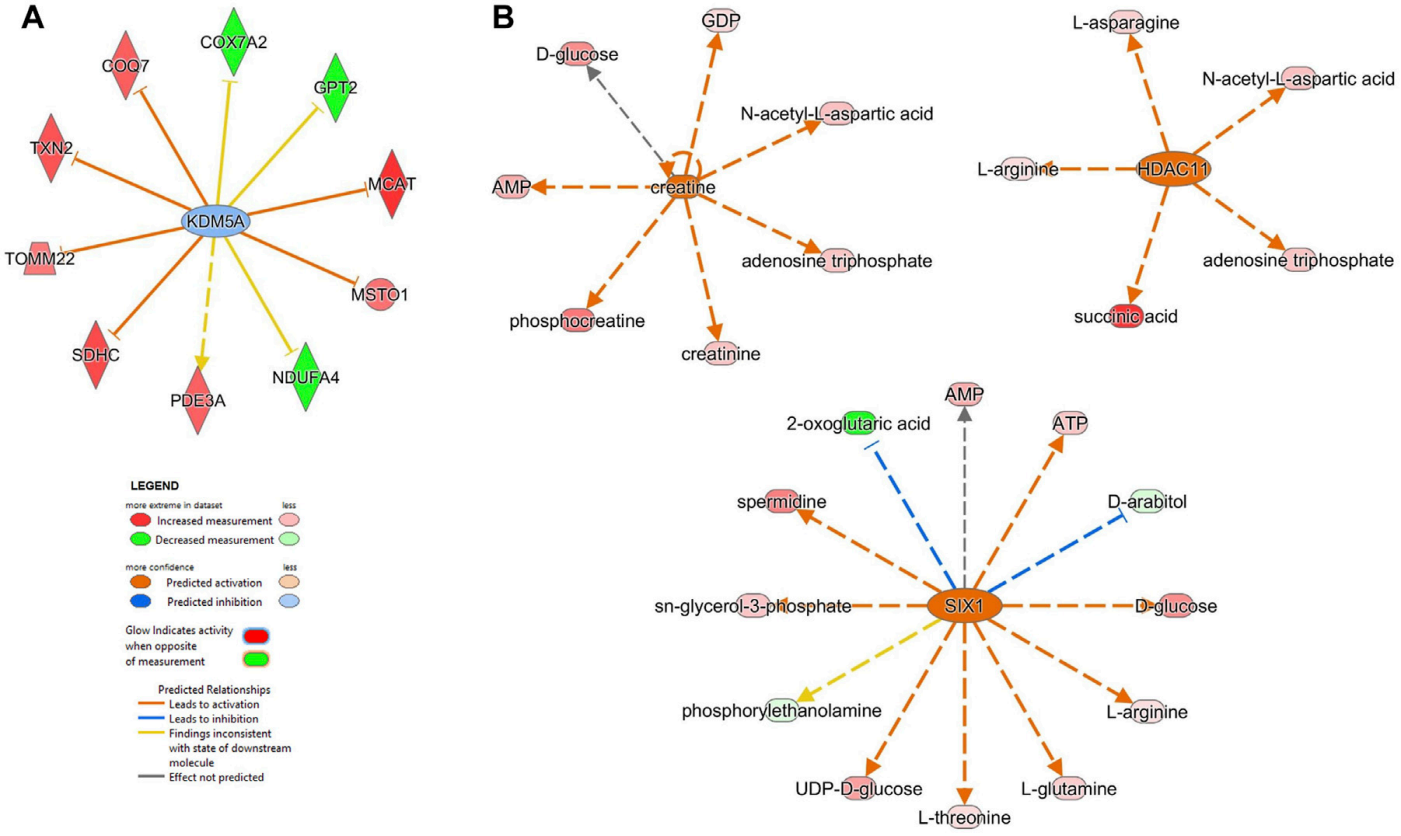
Upstream analysis using proteomics and metabolomics datasets showed potential up-regulators mediating the effects of the enzymatic depletion of mitochondrial polyP in SH-SY5Y MitoPPX cells. **(A)** The proteomics analysis showed that KDM5A (lysine-specific demethylase 5A) is one of the first 10 upstream regulators, which is predicted to regulate different mitochondrial proteins such as COX7A2 subunit of complex IV and NDUFA4 subunit of complex I. **(B)** The metabolomics analysis identified creatine, SIX1 (homeobox protein SIX1) and HDAC 11 (histone deacetylate 11) as upstream molecules predicted to regulate different mitochondrial metabolites such as ATP, AMP, phosphocreatine, succinic acid and 2-oxoglutaric acid. This figure was prepared using IPA predictions from the same samples used for the metabolomics and proteomics assays (five replicates, collected on two independent days).

From 308 predicted upstream regulators of different molecule types identified in the proteomics analysis, only 11 were further considered based on FDR<0.05 (Table 3 and Supplementary Material S1). The transcriptional regulator KDM5A (lysine-specific demethylase 5A), a histone demethylase, was increased in MitoPPX cells within our data set, compared to Wt (log_2_FC = 0.203) and appears as one of the predicted upstream regulators (negative z-score = -0.632, FDR<0.05) of ten proteins, three of them being dysregulated subunits of the ETC complexes in MitoPPX cells: COX7A2-complex IV, NDUFA4-complex I, and SDHC-complex I (Figure 5A). In addition, KDM5A was a predicted upstream regulator of two other mitochondrial proteins: Txn2, a mitochondrial-specific thioredoxin, and Misato 1 (Msto 1), which is a cytosolic protein, involved in the regulation of mitochondrial distribution, morphology, fusion, and network formation (Kimura and Okano, 2007; Gal et al., 2017; Nasca et al., 2017) (Figure 5A).

**TABLE 3:** Analysis of upstream regulators generated from protein changes between MitoPPX and Wt cells identified using IPA software. The 11 predicted upstream regulators with an FDR set at 0.05 are listed below. The activation z-score can be used to infer likely activation states of the upstream regulators based on the direction of protein abundance change in the dataset, i.e. a negative activation z score indicates that the upstream regulator is downregulated in MitoPPX cells compared to Wt. NP indicates no prediction of activation status was generated by the IPA software.

Upstream Regulator	Activation z-score	FDR	Target molecules in dataset
APBB1	0.762	5.97E-03	ACTA2, EGFR, TAGLN, TYMS, VLDLR
CST5	−2.828	5.97E-03	ADSL, AHNAK, BRIX1, EXOC3, MALSU1, MSN, PDCL3, PITRM1, PPAN, PRKACB, ACTA2, EGFR, VIM
GLIPR2	NP	1.66E-02	ACTA2, EGFR, VIM
HNF4A	0.751	1.66E-02	AAMDC, ABCF3, ACTA2, AHNAK, ARFGAP1, ARFIP2, AS3MT, ASNS, C11orf58, CCDC25
miR-382-5p (miRNAs w/seed AAGUUGU)	NP	2.37E-02	SEPTIN3, TAGLN, VIM
MIR143-145a	NP	2.37E-02	ACTA2, DES, TAGLN
KDM5A	−0.632	4.31E-02	COQ7, COX7A2, GPT2, MCAT, MSTO1, NDUFA4, PDE3A, SDHC, TOMM22, TXN2
XAV939	NP	4.31E-02	AHNAK, CSRP1, DES, DHX36, IGFBP2
TP53	1.751	4.31E-02	A2M, ACOT11, ACTA2, ALDH1B1, ASNS, ATG7, BICD2, BID, CHEK1, CNN2
MED28	NP	4.85E-02	ACTA2, CNN2, TAGLN
miR-145-5p (and other miRNAs w/seed UCCAGUU)	−1.851	4.96E-02	ACTA2, AHNAK, C11orf58, CCDC25, NDUFA4, TAGLN, UNG

The metabolomics analysis resulted in 1,013 upstream regulators, with 69 FDR<0.001 (Supplementary Material S1). Creatine, an endogenous compound in neuronal cells which is also involved in bioenergetics, was decreased in MitoPPX cells within our data set, compared to Wt (log_2_FC = -0.019). This metabolite was predicted to be an upstream regulator of seven metabolites (positive z-score = 2.383), including phosphocreatine, AMP and ATP (Figure 5B). In addition, SIX1 (Homeobox protein SIX1) and HDAC 11 (histone deacetylate 11) were predicted as upstream regulators (positive z-score = 2.714 and 2.200, respectively). SIX1 is a transcription factor predicted to lead to decreased levels of 2-oxoglutaric acid and the increase of AMP and ATP (Figure 5B). Also, HDAC11 is predicted to lead to increased succinate levels (Figure 5B).

#### 3.2.5 Analysis of Diseases and Functions

**FIGURE 6:**
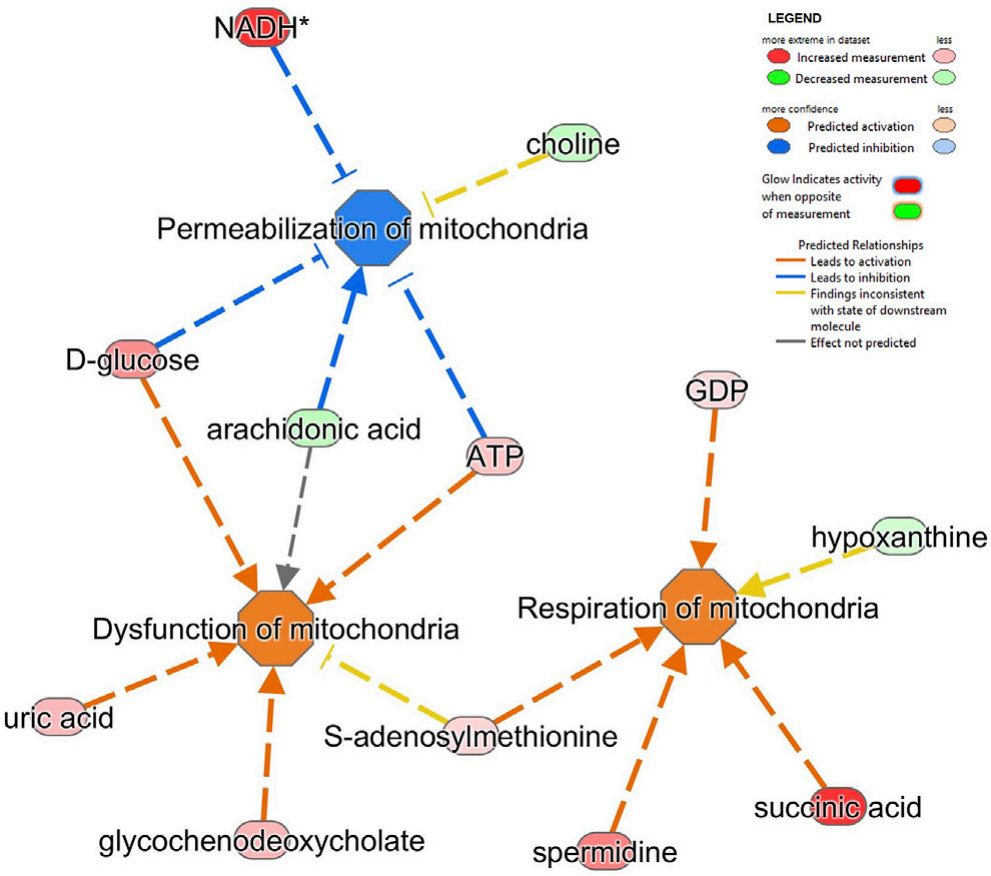
Alterations in several metabolites identified impacts on different mitochondrial pathways in SH-SY5Y MitoPPX cells. Respiration of mitochondria, dysfunction of mitochondria and permeabilization of mitochondria (all of them FDR<0.001) were the top three significantly affected mitochondrial-related pathways shown by ‘disease and function’ analysis in IPA software from the metabolomics data.

We also applied the ‘disease and function analysis’ from IPA to examine the biological context of proteomics and metabolomics alterations in MitoPPX and Wt cells (Supplementary Material S1). In our proteomics analysis, the top five significant diseases or functions were cancer (raw *p* value range: 4.8E-29 - 5.46E-03), organismal injury and abnormalities (raw *p* value range: 4.8E-29 - 5.46E-03), endocrine system disorders (raw *p* value range: 2.98E-20 - 4.12E-03) gastrointestinal disease (raw *p* value range: 2.17E-13 - 4.78E-03); and DNA replication, recombination, and repair (raw *p* value range: 1.1E-07 - 2.56E-03). Cancer was one of the top results. In fact, the role of polyP in carcinogenesis has already been demonstrated (Wang et al., 2003; Tsutsumi et al., 2017; Arelaki et al., 2018; Kulakovskaya et al., 2018). Neurological disease is within the top ten affected ‘disease and functions’, showing 283 proteins involved. In our metabolomics analysis, the top five significant ‘disease or functions’ are cellular growth and proliferation (raw *p*-value range: 5.27E-23 - 2.74E-03), organismal development (raw *p*-value range: 5.27E-23 - 2.74E-03), inflammatory disease (raw *p*-value range: 1.02E-15 - 2.74E-03), inflammatory response (raw *p*-value range: 1.02E-15 - 2.74E-03), organismal injury and abnormalities (raw *p*-value range: 1.02E-15 - 2.74E-03). Within all the detected ‘disease and function’, ten of them were related to mitochondria and affected in neurodegeneration. The most significant included dysfunction of mitochondria, permeabilization of mitochondria, and respiration of mitochondria (all of them FDR<0.001; Figure 6).

### 3.3 Meta-Analysis Corroborates Proteomics and Metabolomics Findings

**FIGURE 7:**
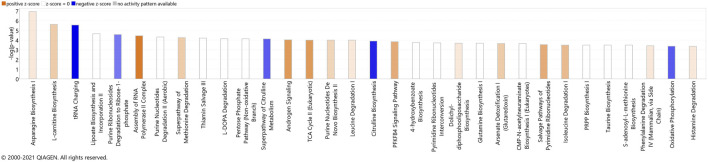
Canonical pathways identified using IPA analysis, based on the meta-analysis. The bars represent the significance of the canonical pathway as calculated by a right-sided Fisher’s exact test. Therefore, the tallest bars represent the canonical pathways that are the least likely to have been identified due to molecules being in the canonical pathway by random chance. Canonical pathways which are likely activated (based on the pattern of differentially abundant proteins or metabolites) are presented in orange (positive z-score) and pathways that are likely inhibited are presented in blue (negative z-score).

**FIGURE 8:**
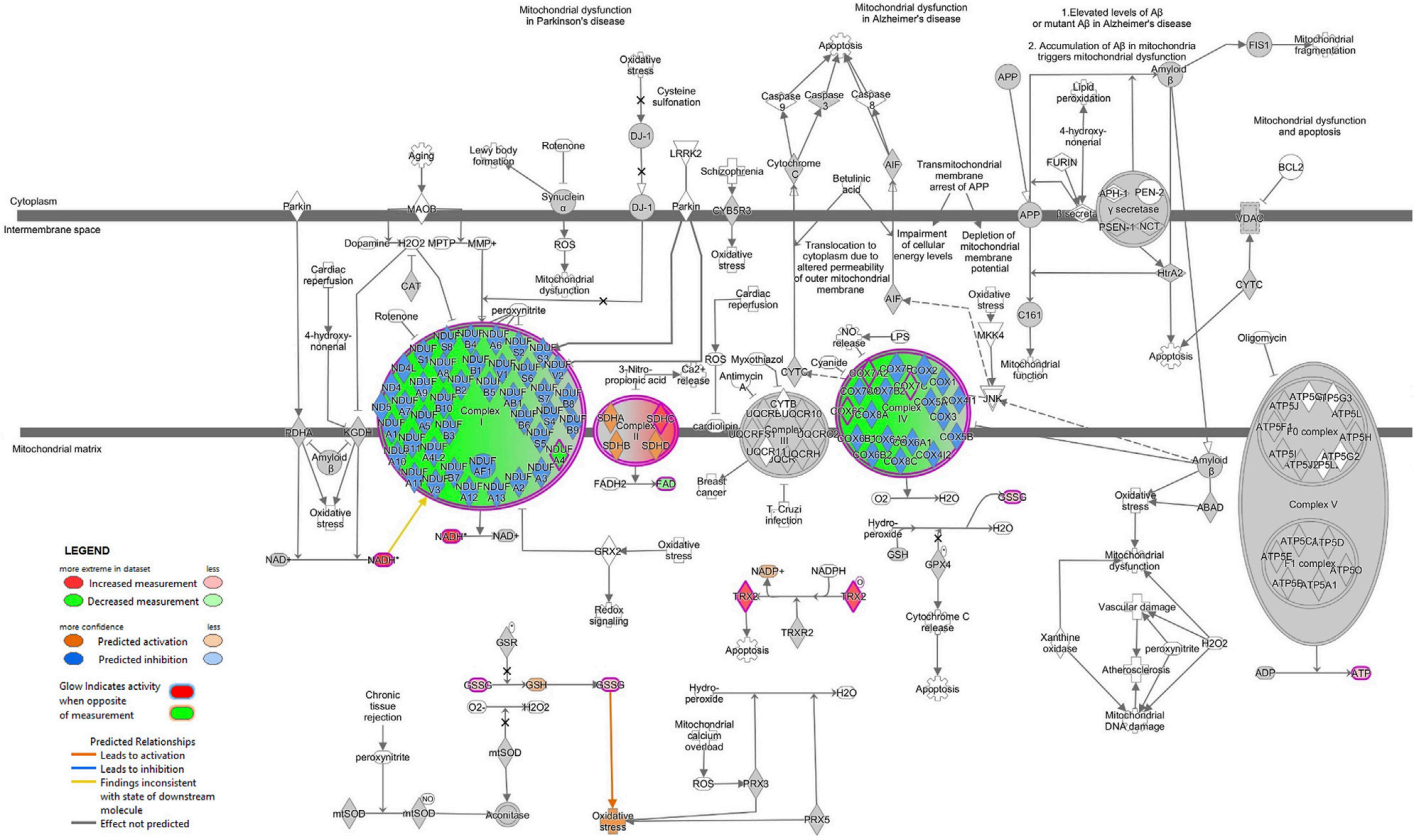
Mitochondrial dysfunction is predicted in SH-SY5Y MitoPPX cells (meta-analysis). Predicted up- and downstream pathways of molecules participating in mitochondrial function were performed using the ‘MAP’ option in IPA software. The decrease of some of the subunits of ETC complex I and IV together with an increase of some of the subunits of ETC complex II lead to an unbalanced functioning of OXPHOS. The prediction states an increase in the glutathione (GSH) and NADP + levels (due to increase in GSSG and TRX2, respectively), probably as an attempt to counteract the potential oxidative stress consequence of a potential mitochondrial dysfunction.

Next, we combined the proteins and metabolites differentially expressed together into an IPA analysis. The meta-analysis of both proteomics and metabolomics assays suggested altered canonical pathways associated with abundant metabolites and proteins in MitoPPX cells (Supplementary Material S1). The most significant pathways found included asparagine biosynthesis I, l-carnitine biosynthesis, tRNA charging, lipoate biosynthesis and incorporation II, and purine ribonucleosides degradation to ribose-1-phosphate (all of them raw *p*-value<.001). The meta-analysis further supported the decreased OXPHOS pathway in MitoPPX cells (negative z-score = -1.76, Figure 7), observed in both the proteomics and metabolomics study, as well as the change in TCA (positive z-score = 2.00, Figure 7) observed in the metabolomics analysis. In addition, the meta-analysis revealed the presence of mitochondrial dysfunction in MitoPPX cells, when compared with Wt samples (grey z-score = NaN, Table 4). Using the ‘Molecule Activity Predictor’ (MAP) option in IPA software, we predicted up- and downstream properties/activities of molecules participating in mitochondrial function (Figure 8). These included an increase in the glutathione levels (GSH), probably to counteract the potential increased oxidative stress in MitoPPX cells, as well as an increase of TRX2, which is a mitochondrial protein important for the control of mitochondrial ROS homeostasis, leading to an increase of NADP + levels (Figure 8).

**TABLE 4:** List of the proteins and metabolites found altered in the mitochondrial dysfunction pathway in MitoPPX cells compared to Wt (meta-analysis conducted by IPA software). Table shows increase (log_2_FC > 0) or decrease (log_2_FC < 0) of different proteins and metabolites involved in the canonical pathway of mitochondrial dysfunction in the meta-analysis. FDR: False Discovery Rate.

Symbol	Uniprot/Human Metabolome Database (HMDB)	Log2FC	FDR
Adenine-riboflavine dinucleotide	HMDB01248	−1.13	7.78E-05
ATP	HMDB00538	0.91	2.69E-02
COX6C	P09669	−2.67	2.40E-21
COX7A2	P14406	−2.31	1.89E-21
COX7C	P15954	−1.54	2.78E-07
Glutathione disulfide	HMDB03337	0.99	2.54E-03
NADH	HMDB01487	4.70	8.55E-06
NDUFA4	O00483	−2.00	3.82E-17
SDHC	Q99643	2.61	3.38E-06
TXN2	Q99757	2.45	1.76E-09

Moreover, we identified 57 different upstream regulators with FDR set at 0.05 (Supplementary Material S1). The analysis distinguished 40 regulators as genes, RNAs or proteins, such as the previously mentioned SIX1 (activation z-score = 2.714, FDR<0.001) and HDAC11 (positive z-score = 2.200, FDR<0.01). Additionally, IPA predicted another upstream regulated protein, UCP1 (uncoupling protein one; positive z-score = 2.027, FDR<0.01).

Finally, the meta-analysis showed top five most significant dysregulated pathways as cancer (raw *p* value range: 1.66E-18–2.95E-03), organismal injury and abnormalities (raw *p* value range: 1.66E-18–2.95E-03), cellular growth and proliferation (raw *p* value range: 8.04E-13–2.44E-03), organismal development (raw *p* value range: 8.04E-13–2.68E-03), and endocrine system disorders (raw *p* value range: 2.77E-11–2.68E-03). In line with metabolomics data, we identified several significantly affected mitochondrial-related pathways, such as dysfunction of mitochondria, release of mitochondrial DNA, respiration of mitochondria, and activation of mitochondria (all of them FDR<0.05; Supplementary Material S1).

## 4 Discussion

A wide range of mitochondrial alterations have been described in the etiopathology of various human diseases. These alterations include but are not limited to dysregulation of the ETC complexes, which will ultimately affect OXPHOS functionality, increasing ROS levels and unbalancing the cellular antioxidant system (Guzun et al., 2011; Gonzalez-Casacuberta et al., 2018; Gonzalez-Casacuberta et al., 2019). However, the exact molecular basis of the mechanism(s) underlying mitochondrial dysfunction still remain mostly unknown.

PolyP has been described as a key molecule in the cellular stress response, including the regulation of several mitochondrial functions, such as energy metabolism (including calcium homeostasis), and protein homeostasis (Gray et al., 2014; Dahl et al., 2015; Gray and Jakob, 2015; Solesio et al., 2016a; Cremers et al., 2016; Lempart and Jakob, 2019; Xie and Jakob, 2019; Solesio et al., 2020; Solesio et al., 2021). However, the exact molecular mechanism explaining the effects of polyP in the regulation of mitochondrial function are still unclear, especially under stress conditions, such as in human disease.

To our knowledge, this is the first time that a comprehensive study of the effects of polyP on the proteome and the metabolome of mammalian cells has been conducted. Specifically, in the present study, a large number of protein expression and metabolite abundance alterations were described in SH-SY5Y cells that were enzymatically depleted of mitochondrial polyP (MitoPPX). These findings highlighted the importance of polyP in maintaining the proper cellular function, either through direct or indirect regulation of all proteins and metabolites that are differentially expressed in MitoPPX cells.

The number of differentially expressed mitochondrial proteins and the different abundance of mitochondrial metabolites observed in MitoPPX cells showed the clear impact that enzymatical depletion of mitochondrial polyP has in the metabolism of the organelle. Two of the main dysregulated mitochondrial metabolic pathways identified by IPA software were OXPHOS and TCA. In our case, when we used IPA, the background was set for all proteins identified in the samples and not for the entire protein coding transcriptome (default in IPA). Specifically, ETC complex I, complex II, and complex IV showed differential protein expression of some of their subunits, leading to a potential alteration of their activity. Moreover, altered levels of succinate and oxoglutarate evidenced the dysregulation of the TCA. Indeed, the observed increased succinate ETC complex II subunits could indicate a blockage of this complex. In addition, further evidence that demonstrated that mitochondrial metabolism was affected, were increased levels of ATP, ADP, NAD, and NADH in MitoPPX cells. These findings reinforced the importance of polyP in the maintenance of mitochondrial and cellular function. One plausible mechanism explaining this differential pattern of protein and metabolites presence in MitoPPX cells could be the reduced chaperoning effects due to the lack of polyP in our model (Gray et al., 2014; Gray and Jakob, 2015; Lempart and Jakob, 2019). It is well known that the chaperoning ability of the cells is critical in the activation of the stress response. Indeed, the lack of mitochondrial polyP could promote the activation of the cellular stress response in MitoPPX cells. This explanation is supported by our proteomic analysis data, showing increased expression of some antioxidant enzymes (including several peroxiredoxins, thioredoxins, SOD1 and catalase), as well as of proteins involved in the regulation of the cellular stress response (including KEAP, Kelch-like ECH-associated protein 1). This entire cellular stress environment could further deleteriously affect mitochondrial metabolism.

The observed effects of polyP could also be at least partially mediated by the direct interaction between the polymer and the altered proteins within the OXPHOS and TCA which were identified in our analysis. In contrast to the mentioned increased expression of several antioxidant enzymes, mitochondrial SOD2 expression was decreased in MitoPPX cells, which hints at this enzyme as a potential target of polyP. Mitochondrial SOD2 is a superoxide dismutase enzyme dependent on manganese, which is known to form complexes with polyP (Gray and Jakob, 2015). This property suggests a role for polyP in the detoxification process mediated by SOD2. Although we did not show direct evidence of increased ROS levels in these MitoPPX cells, the regulatory effects of the polymer on ROS have already been demonstrated in other organisms (Gray and Jakob, 2015). As previously mentioned, our data showed that ETC complexes I and IV were affected in MitoPPX cells. Some authors have evidenced an overall alteration of oxygen consumption as a consequence of these deficiencies in the ETC, which was also demonstrated by previous work, which was conducted in HEK293 cells (Solesio et al., 2021).

Thanks to our analysis, we were able to identify several upstream regulators of these changes in IPA analysis. For example, KDM5A was identified to regulate mitochondrial proteins, confirming that the lack of polyP could be involved in the decrease of KDM5A leading to the decrease of ETC subunits (COX7A2-complex IV and NDUFA4-complex I), and the increase of catalase and TXN2 (both participating in the antioxidant defense). KDM5A is a well-known regulator of the expression of mitochondrial proteins, especially of those involved in mitochondrial respiration (Varaljai et al., 2015). Furthermore, SIX1, a transcriptional factor described to regulate mitochondrial apoptosis (Du et al., 2017), was predicted to lead to decreased levels of 2-oxoglutaric acid and increased ATP levels. These alterations could be the potential cause underlying the TCA abnormalities observed in MitoPPX cells. Finally, creatine was identified to upregulate ATP and phosphocreatine among others. Creatine is an endogenous chemical converted into phosphocreatine, which is used to produce new ATP under certain circumstances (i.e. during high-intensity exercise) (Guzun et al., 2011). Moreover, the protective role of creatine in maintaining mitochondrial physiology is also known (Barbieri et al., 2016), and it has been demonstrated that the dietary supplementation with this molecule could improve cellular bioenergetics and mitochondrial function, therefore decreasing neuronal cell death in neurodegeneration (Adhihetty and Beal, 2008; Smith et al., 2014). In addition, IPA predicted three upregulated proteins, SIX1, UCP1, and HDAC 11, all of them involved in the upstream regulation of mitochondrial proteins and metabolites, as well as mitochondrial bioenergetics (Fedorenko et al., 2012; Kazak et al., 2015; Chowdhury et al., 2017; Yang et al., 2017; Bhaskara, 2018; Hurtado et al., 2021). In general, the ‘upstream regulators’ analysis hinted at a cellular compensatory response in MitoPPX cells, in which increased presence of proteins involved in cell stress response, including ATP generation, was present, when compared with the Wt samples.

This study has some limitations. We recognize that the use of geneticin exclusively in the MitoPPX cells could exert some effects on the physiology of the SH-SY5Y cells. Additionally, transfection with the MitoPPX contrast could change the amount and/or type of proteins in mitochondria. Moreover, our data was not sufficient to clarify the exact molecular mechanisms by which mitochondrial polyP was involved in the regulation of bioenergetics within the organelle and therefore, in the regulation of mitochondrial physiology. Indeed, the present proteomics ad metabolomics approaches were not focused on the investigation of these mechanisms but to obtain a snapshot of mitochondrial metabolism. Further research is needed to better understand the role of polyP within mitochondria, especially in the context of neurodegeneration. However, this proteomics and metabolomics analysis bring new insights towards elucidating the role of polyP in the regulation of mitochondrial physiology in mammalian cells. Moreover, targeting the metabolism of polyP could provide us with novel therapeutic approaches for diseases where mitochondrial dysfunction has been broadly described as an early and triggering event, such as in neurodegenerative disorders.

## Data Availability

Further information and requests for resources and reagents should be directed to and will be fulfilled by the corresponding author, MS (m.solesio@rutgers.edu). Proteomics data (skyline documents and raw files for DIA library generation and DIA sample analysis) has been deposited in Panorama Public (ProteomeXchange ID: PXD028185. Access URL: https://panoramaweb.org/MitoPPX.url). The full data set from the metabolomics assay can be found at Supplementary Information.
