# Supplementary figures and images for: Mitochondrial Inorganic Polyphosphate (polyP) Is a Potent Regulator of Mammalian Bioenergetics in SH-SY5Y Cells: A Proteomics and Metabolomics Study
Source: Front Cell Dev Biol. 2022 Feb 17;10:833127. doi: 10.3389/fcell.2022.833127 (PMC8892102; doi:10.3389/fcell.2022.833127)

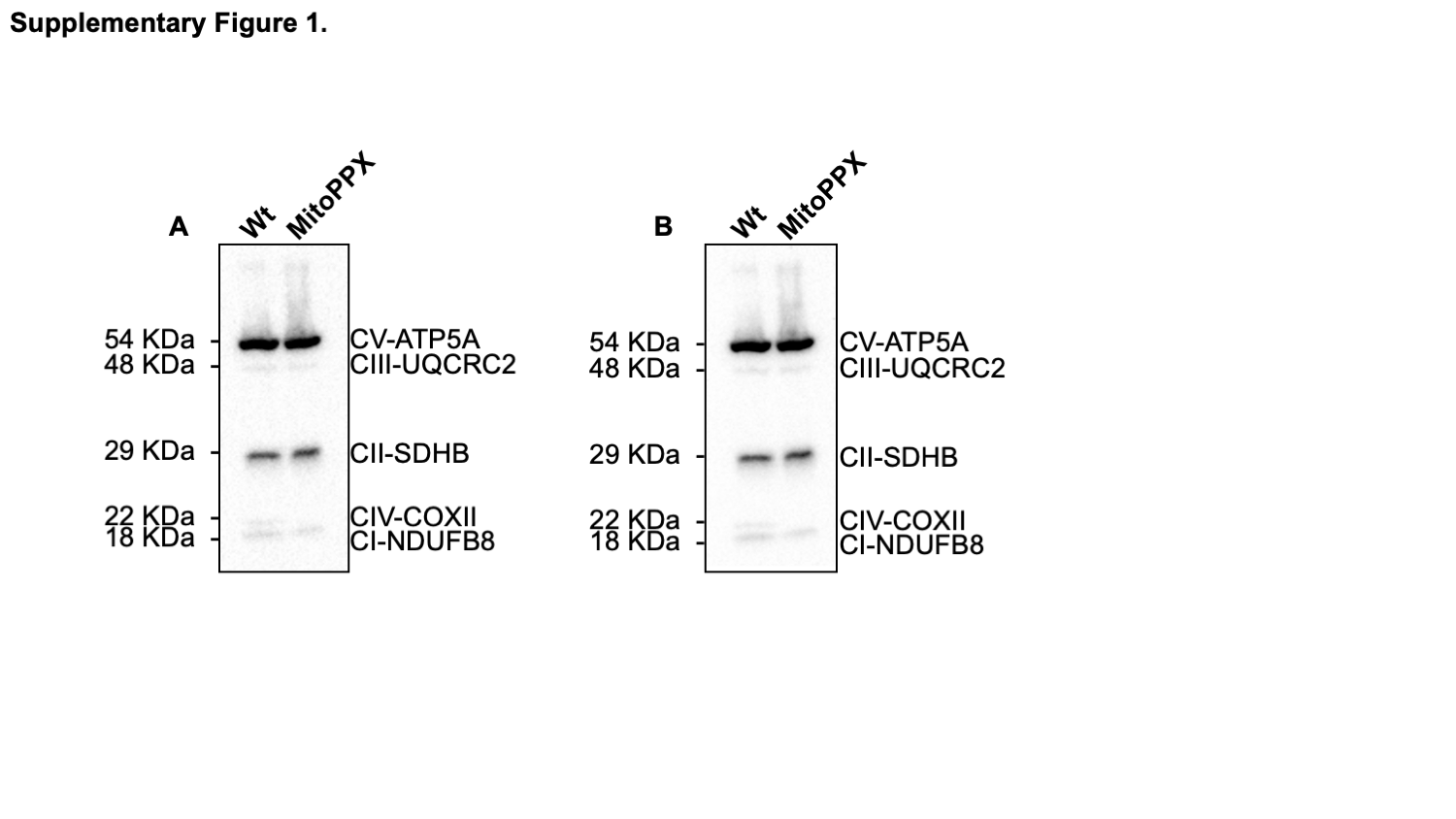

Supplement: Supplementary file 2 [file Image1.TIFF]

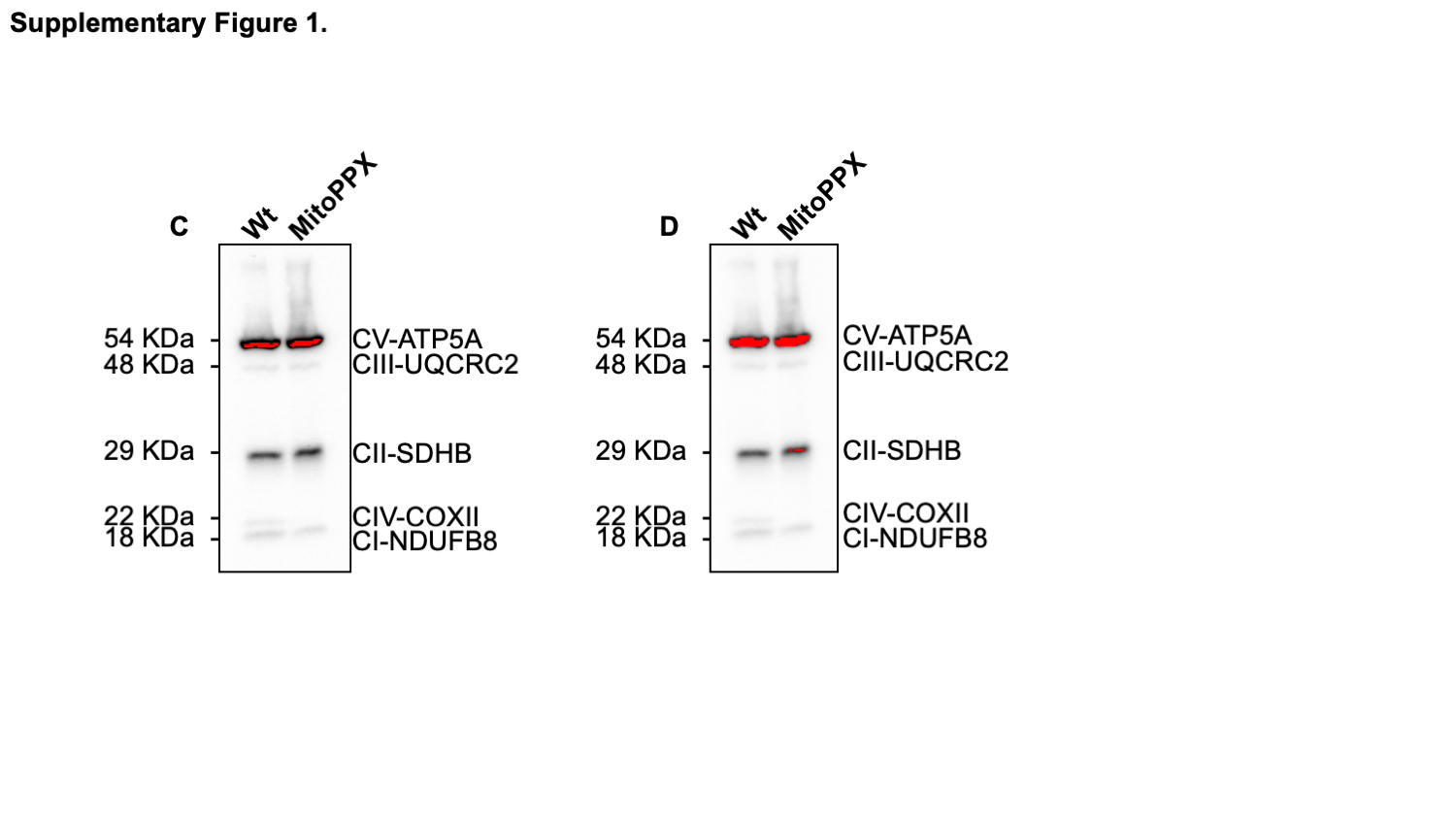

Supplement: Supplementary file 6 [file Image2.TIFF]
